# Investigation of the effects of starch on the physical and biological properties of polyacrylamide (PAAm)/starch nanofibers

**DOI:** 10.1007/s40204-017-0069-7

**Published:** 2017-07-26

**Authors:** Hadi Taghavian, Seyed-Omid Ranaei-Siadat, Mohammad Reza Kalaee, Saeedeh Mazinani

**Affiliations:** 1 Department of Polymer Engineering, Islamic Azad University, South Tehran Branch, Tehran, 1777613651 Iran; 2 Nanobiotechnology Engineering Laboratory, Department of Biotechnology, Faculty of Energy Engineering and New Technologies, Shahid Beheshti University, GC, Tehran, Iran; 3 Amirkabir Nanotechnology Research Institute (ANTRI), Amirkabir University of Technology, Tehran, Iran

**Keywords:** Starch, Nanofibers, Polyacrylamide, Enzyme immobilization, Biological property

## Abstract

Here, we report the development of a new polyacrylamide (PAAm)/starch nanofibers’ blend system and highlight its potential as substrate for efficient enzyme immobilization. PAAm was synthesized and blended with starch. The final blend was then electrospun into nanofibers. The response surface methodology was used to analyze the parameters that control nanofiber’s diameter. Electrospun mat was then modified either by cross-linking or phytase immobilization using silane coupling agent and glutaraldehyde chemistry. Physico-chemical properties of blends were investigated using spectroscopic and thermal studies. The evaluation of immobilized enzyme kinetics on both pure and the starch blended PAAm nanofibers was performed using Michaelis–Menten kinetic curves. Fourier transform infrared spectroscopy results along with differential scanning and X-ray diffraction confirmed that blending was successfully accomplished. TGA analysis also demonstrated that the presence of starch enhances the thermal degradability of PAAm nanofibers. Finally, it was shown that addition of starch to PAAm increases the efficacies of enzyme loading and, therefore, significantly enhances the activity as well as kinetics of the immobilized enzyme on electrospun blend mats.

## Introduction

There has been a great progress in the preparation, function, and evaluation of biomaterials concerning multifaceted practicability of them. Thanks to the outstanding properties and the key role in diversified areas of performance, a wide range of biomaterials has been increasingly used in biological applications. The acknowledgement that investigation of the impact of natural biopolymer-biomaterial along with its relevant mechanical and biological experiments comprising enzymatic survey can be amply efficient stimulates the authors to pursue this novel subject.

Chemical and physical properties of starch have been widely studied due to its flexibility for a variety of applications because of its biodegradability and economical availability (Whistler et al. 1984; Mano et al. 2003; Kaushik et al. 2010). The advantage that glycosidic bonds between monosaccharides provide polysaccharides with good biocompatibility and availability properties makes it suitable to be used as cell carriers in tissue engineering (Bačáková et al. 2014). Polysaccharides are even able to improve mechanical properties of natural fibers (Curvelo et al. 2001; Kaushik et al. 2010). It was observed that various blends of polysaccharide component show good potential to be used in numerous biomedical applications (Shelke et al. 2014). Polysaccharides such as starch modified nanofibrous scaffolds are capable of promoting cellular activities through increasing bioactivity and cell retention (Bačáková et al. 2014). Biocompatibility and cell cultural properties of scaffolds obtained from blended starch nanofibers can significantly intensify the cells viability (Gomes et al. 2006; Salgado et al. 2004). Starch-based scaffolds can diminish diffusion constraints and mechanically stimulate the marrow stroma cells to develop the bone-like mineralized tissue (Gomes et al. 2006). Gomes et al. (2006) showed that starch-based scaffolds affect not only the sequential development of osteoblastic cells but also the functionality of tissues formed in vitro. To our knowledge, the role of natural polysaccharide nanofibers as a bed for enzyme immobilization and its effects on physical and biological properties has not been thoroughly investigated. So here, we aim to study the role of starch in blended nanofibers and further study its potential ability on improving enzymatic performance.

Electrospinning is such a commonly used, convenient, versatile, and economically effective process that creates electrified jets from various polymer solutions including even polymer blends, composites, etc. (Ramakrishna et al. 2005). In this process, the solution is contained in a capillary with a syringe tip and a collecting device. Nanofibers will be stretched out from the solution by applying high voltage to the polymer jet (Ramakrishna et al. 2005). Nanofibers have many remarkable advantages and superior mechanical properties in comparison with other fibers. The properties of nanofibers including their very large surface area-to-volume ratio and porous morphology in nanoscale range make them feasible to be employed in numerous applications such as in drug delivery and tissue engineering or as a nanofibrous mat and a proper bed for enzyme immobilization (Ramakrishna et al. 2005; Amini et al. 2013; Lee et al. 2005; Mohebali et al. 2016; Shelke et al. 2016; Oktay et al. 2015; Leung and Ko 2011).

Enzyme immobilization on nanofibrous mat not only does reform instability and non-reusability of the enzyme, but also enhances enzyme’s reaction time which is an efficient way to eliminate its defects (Amini et al. 2013; Wang et al. 2009; Wagner et al. 2015). Efficacy of enzyme such as: biocompatibilities, etc., entirely depends on the bed employed for immobilization (Amini et al. 2013; Wang et al. 2009; Amini et al. 2012; Ebadi et al. 2015). Of many prominent biological properties of nanofibrous nanowebs is its ability to be biodegradable by microorganisms. It is supposed that starch compounded nanofibers make a prospective candidate as a bed in biological applications and enzyme immobilization (Shelke et al. 2014).

Fabrication of nanofibers out of pure starch and without blending with other synthetic high-molecular-weight polymers is very challenging. Here, we used the blending technology to prepare multi-functional nanofibers to meet our specific objectives. We chose to work with polyacrylamide (PAAm) which is one of the most important superabsorbent polymers. PAAm has a very routine synthesis protocol and it is known as one of the economical biocompatible polymers. PAAm was selected to be compounded with starch. It is supposed that its ability to absorb a large amount of water to form a stable hydrogel makes PAAm a promising candidate for developing biological applications and immobilization of aqueous enzyme’s solutions (Amini et al. 2013).

We employed design of experiments (DOE) method to investigate the effect of addition of starch (interconnected factor) on the diameter of PAAm nanofibers (selected response). DOE fits experimental results to mathematical equations representing some models based on a combination of values to predict the process (Chow and Yap 2008). Response surface methodology (RSM) is an empirical-based technique of gathering statistical and mathematical models to design, model, and analyze experiments (Chow and Yap 2008; Montgomery 1997; Yalcinkaya et al. 2010; Bas and Boyac 2007; Yördem et al. 2008; Rabbi et al. 2012). RSM can be used to determine optimum operating conditions by detecting the relation of an influenced objective response and several variables (Chow and Yap 2008; Montgomery 1997; Yalcinkaya et al. 2010; Bas and Boyac 2007; Yördem et al. 2008; Rabbi et al. 2012). It determines the effect of independent variables on a selected process (Yördem et al. 2008; Heydari et al. 2013). Therefore, RSM is utilized to investigate the effects of PAAm concentration and starch content on nanofibers’ diameter. The aim of this study is to investigate the impact of starch on the process ability and characterization of physical and biological properties of native starch-based nanofibrous systems.

## Experimental

### Materials

Acrylamide monomer (AM), (71.08 g/mol, Merck) was used for polyacrylamide (PAAm) synthesis. Potassium peroxodisulfate (KPS), (270.33 g/mol, Merck) was used as a radical initiator for the synthesis reaction of PAAm. 3-Aminopropyltriethoxysilane (APTES) and glutaraldehyde (GA) were bought from Sigma-Aldrich Co and PanreacQuimica San Co. for cross-linking reaction of nanofibers, respectively. Wheat starch was a general food grade prepared from the market. Formic acid was purchased from Riedel–de Haen as a solvent for electrospinning. Sodium phytate (P3168) was bought from Sigma-Aldrich Co. as a substrate of the phytase. *Escherichia coli* DH5α and primers for PCR were purchased from Sinaclone Co. The sequence encoding *Aspergillus niger* Phytase (P34752) was synthesized by ShineGene Molecular Biotech (Shanghai, China). Other analytical grade chemicals were obtained from Merck Co.

### Experimental design

The experiments were conducted to determine the role of adding starch in controlling the morphological properties of nanofibers. The effects of polymer weight fraction and blended starch percentage as independent variables and functional relationship between them attributed to molecular weight, viscosity, conductivity, and surface tension on the morphology of nanofibers were studied using RSM. Experimental designs were carried out by choosing the range of the weight fraction of polymer from 1.5 to 4 wt% and an appropriate proportion of blended starch from 20 to 50%. The central composite design (CCD) was used as an experimental design to obtain equal prediction in all directions from the center in which the variables vary in 5 levels with 5 axial points and 5 replicates at the center. Totally, it was indicated that 13 experiments were required to fit a second-order polynomial model (Table 1).

**Table 1:** Experimental design and resulted responses

Experiment no.	wt% of PAAm (g/ml)	Starch compounding (%)	Average fibers diameter (nm)
1	2.75	35	206.1 ± 15
2	2.75	20	218.3 ± 18
3	3.63	24.39	241.2 ± 17
4	1.87	24.39	144.9 ± 15
5	2.75	35	208.7 ± 20
6	2.75	35	218.3 ± 12
7	2.75	50	200.7 ± 16
8	4	35	242.0 ± 25
9	2.75	35	201.7 ± 14
10	1.87	45.61	122.1 ± 10
11	1.5	35	120.2 ± 14
12	3.63	45.61	230.7 ± 20
13	2.75	35	206.0 ± 19

### Polyacrylamide synthesis

50 g of acrylamide was dissolved in 200 ml distilled water. The solution was then air-degassed and heated up to 80 °C beneath nitrogen atmosphere. Radical initiator and oxidant, KPS (0.5% wt concentration to acrylamide), was added to the solution and free radical polymerization was started after initiator was added to the monomers. After 2 h, the polymerization was stopped and the resultant product was precipitated with acetone and preserved in vacuum desiccators.

### Electrospinning

We made starch/PAAm solutions according to the experimental design (Table 1) by dissolving starch and PAAm separately in formic acid. Solutions were heated up to 50 °C and stirred for 3 h. Then, they were mixed up together and stirred for 48 h at room temperature. The polymer concentration was varied from 1.5 to 4% (w/v, with respect to the solvent) and the percentage of added starch changed from 20 to 50% (w/w, with respect to the polymer concentration). The polymer blend solutions were injected into 1 ml syringe which was fixed horizontally on a syringe pump (model: JMS SP-500). The flow rate of the solutions was adjusted at 0.1 ml/h. The electrode of the high voltage–power supply (model: Gold Star PZ-121) was clamped to the metal needle tip and a 15 kV voltage was applied to generate the electrical field. The tip-to-collector distance was adjusted to 20 cm. To spin the solutions, the functional parameters: feed rate, applied voltage, and tip-to-collector distance were verified, and the uniform nanofibers were obtained. A 100 cm^2^ grounded static vertical collector covered by a piece of aluminum foil was used for the fiber deposition. The complete electrospinning apparatus was enclosed under a vacuum hood and an electrospinning process was carried out at room temperature.

### Cloning and expression of phytase

The recombinant vector PUC57 containing *Aspergillus niger* Phytase was transformed to the competent cell of *E*. *coli*. After colony PCR screening, plasmids were purified from the positive clones by the alkaline lysis method and then digested with *Hin*dIII/*Bam*hI. The phytase gene was inserted into pFPMTMF*α* as an expression and also as a shuttle vector. pFPMTMF*α* comprising phytase was transformed into *E*. *coli* DH5*α*. All the cloning steps were done according to Sambrook et al. (1989). The phytase was transformed into *Pichiapastoresis*.

The pre-culture was grown in a 250 ml flask containing 50 ml of YPD (1% yeast extract, 2% peptone, and 2% glucose) at 37 °C for an overnight (16–18 h) on a rotary shaker at 250 rpm. The main culture medium (1% yeast extract, 2% peptone, 1% glycerol) was inoculated with 5–10% (v/v) from pre-culture after washing by sterile water. The main cultivation for enzyme production was performed for 72 h under the same conditions as with the pre-culture. The cells were harvested by centrifugation at 2500×*g* for 10 min. The extracellular activity was determined in the cell-free supernatant.

### Phytase activity assessment

We test the phytase assessment by measuring the amount of inorganic phosphate liberated from sodium phytate (Heinonen and Lahti 1981). Different amounts of phytase in a solution were evaluated and 3 U/ml was identified as the optimum phytase concentration in an enzymatic aqueous solution. The assay mixture contains 0.5 ml of 2.5 mM Na-phytate (in 0.1 M pH 5.0 Na-acetate buffer) and 0.475 ml of 0.1 M Na-acetate buffer (pH 5.0). All nanofibers were immersed in the assay mixture and were incubated for 30 min at 50 °C. The reaction was stopped by keeping nanofibers out from the mixture and adding 2 ml of color reagent solution (25% ammonium molybdate solution 5% + sulfuric acid 5 N + acetone). After 45 s, color was developed and 0.1 ml citrate 1 M was added to each test tube to fix the color. Finally, absorbance was read at 380 nm. The activity of phytase before and after immobilization process was assessed to determine the amount of bound enzymes. One unit (1 U) of phytase was defined as the amount of phytase which releases 1 μmol inorganic phosphate per minute at the assay condition.

### Cross-linking and enzyme immobilization

Blended PAAm/starch nanofibers were modified through cross-linking modification to attain biological applications. Of many approaches for cross-linking the surface of nanofibers, we selected an approach to raise stability and to create appropriate links for bonding using APTES and GA, respectively (Amini et al. 2013; Frone et al. 2013). Samples were immersed in the 20 v/v% solutions of APTES in methanol at 50 °C for 4 h and 1 v/v% solutions of the GA in methanol at 70 °C for another 4 h, while shaking, respectively. To remove residual links from non-reacted APTES and GA factors, they were well washed with NaAc buffer which keeps pH constant at 5.0. Through this approach, flexible and appropriate nanofibrous mats were obtained. The nanofibers were immersed in 2 ml synthesized phytase solution at 4 °C overnight while shaking. After removing nanofibers out of enzyme solution, un-attached enzymes were washed three times with NaAc buffer and their activities were assessed.

New blended starch nanofibers retained their physical shape after keeping in the enzyme solution for a long time which endorses high stability after cross-linking. In contrast, nanofibers obtained from pure PAAm nanofibers did not retain their structure without the same cross-linking method, and they were gradually destructed after maintaining in enzyme or buffer solution. This phenomenon can be explained by considering the high hydrophilic nature of PAAm.

### Characterization

The morphology of the electrospun blended PAAm/starch nanofibers was characterized with a high-resolution scanning electron microscopy (SEM) (Hitachi S4160); and ImageJ software was used for measurement of the fibers’ diameter. Design Expert software (version 7, Stat-Ease, Inc., Minneapolis, MN 55413) was used for evaluation of the fibers’ diameter based on the affected nanofibers variables. Fourier transform infrared spectroscopy (FT-IR, model: Nicolet IR100) was used to study differentiations of spectra for starch, PAAm, and blended PAAm/starch which could indicate the changes in the chemical structure upon blending. Thermal analysis of the fibers was done using differential scanning calorimetry (DSC) (NETZSCH 200 F3) and thermal gravimetric analysis (TGA) (Linseis PT-10). DSC measurement allows one to evaluate the thermal behavior differences of the nanofibers and determining the influence of starch on PAAm nanofibers, before and after cross-linking modification. About 11 mg of nanofibers were heated up at 10 °C/min from room temperature to 400 and 800 °C at 10 °C/min under nitrogen atmosphere for DSC and TGA analysis, respectively. Wide angle X-ray diffraction (XRD, model: Siemens D500) measurements were performed to obtain crystalline structure information. The X-ray generator was equipped with an iron tube operating at 35 kV and 25 mA. The wavelength of irradiation was ~0.194 nm. XRD diffractograms were acquired at room temperature over a 2*θ* range of 5°–40° at 1.2°/min. The enzyme activity was evaluated according to Henion and Lathi protocol including some modification. The nanofibrous mat was all prepared at the same size of 1 cm^2^. Optical density (OD) of immobilized phytase mixtures was measured via a UV–Vis Spectrometer (Micro plate reader, model: NanoQuant, infinite M200 Pro) at the absorbance of 380 nm. Michaelis–Menten kinetics for immobilized and free phytase were measured for different phytic acid concentrations as substrate.

## Results and discussion

### Physical properties

#### Morphological analysis

**Fig. 1:**
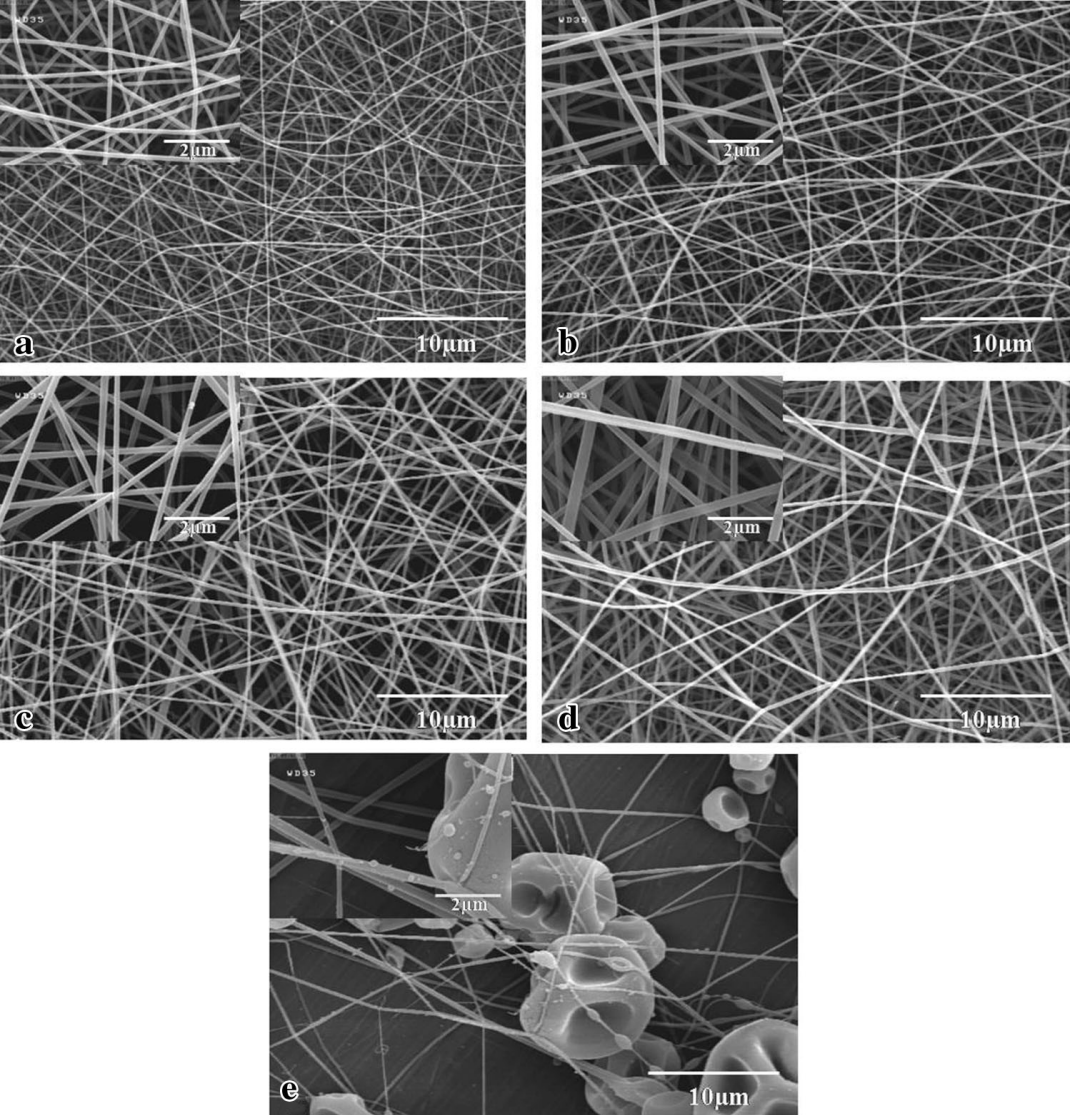
Scanning electron microscopy showing the effect of addition of starch on the PAAm fibers’ diameter: **a** pure PAAm 2.75 wt%, average diameter (ave. dia.): 134.7 nm; **b** 2.75 wt% (50:50) (ave. dia.): 200.7 nm; **c** 2.75 wt% (65:35) (ave. dia.): 206.1 nm; **d** 2.75 wt% (80:20) (ave. dia.): 218.3 nm; **e** wheat starch

SEM images show the random topography of the nanofibers. Influential parameters of the polymer molecular weight, polymer concentration, and polymer solubility in the solvent associate with dependent solution viscosity, conductivity, and surface tension which all directly affect the morphology of electrospun nanofibers (Pham et al. 2006; Daughton 1988). Either increasing polymer concentration or dissolving high-molecular-weight polymer increases the viscosity of the solution and contributes to increasing the diameter of the produced nanofibers (Pham et al. 2006; Bhardwaj and Kundu 2010). Figure 1 shows the SEM images of the PAAm, blended PAAm/starch fibers, and also pure wheat starch. ImageJ software was used to determine the average diameter of the resultant fibers, as shown in Table 1.

As it is shown in Fig. 1e, fabrication of pure starch nanofibers is quite challenging and leads to the bead shape structure. Blending with other synthetic high-molecular-weight polymers such as PAAm is the answer. At a definite weight fraction of the blended PAAm/starch solution, increasing weight fraction of the starch proportionally leads to a reduction in the weight fraction of the PAAm. Subsequently, a reduction of the PAAm concentration in the solution apparently can reduce the viscosity of the solution and can conduce to decline of the nanofibers’ diameter. This can be obviously inferred from pursuing the resultant values of the diameter of a 2.75 wt% solution with a PAAm:starch blending proportion of (80:20) to (50:50), respectively.

#### Diameter analysis via RSM method

Response surface methodology was used to analyze the impact of the addition of starch on the fibers’ diameter (Table 1). The diameter analysis which determines the effect of independent variables, at different levels of weight fraction of the polymer and percentage of the blended starch, is carried out by RSM method (Yördem et al. 2008). Experimental results were analyzed to establish the mathematical functional relations to confirm the variables of the model and they were fitted to a quadratic equation in which the fitness of each term was analyzed by the analysis of variance (ANOVA). Table 2 shows the variance analysis results of the suggested model including corresponding model coefficients and coefficient of determination which imply that quadratic model is significant. The interaction between independent variables and suggested model were inspected by multiple coefficient of determination, *R* ^2^, whose value was found to be 0.9749, and also adjusted coefficient of determination, $R{}^{2}_{\text{Adj}},$ equal to 0.9570 (Chow and Yap 2008). Comparing “calculated coefficient” values of variables demonstrates that the weight fraction of the polymer is the most influential factor on the fibers’ diameter.

**Table 2:** ANOVA results of suggested model

Parameter	*F* value	*P* > *F*	Calculated coefficient	Value
Constant	_	_	208.18	_
*A*	241.01	0.0001	47.15	_
*B*	5.74	0.0477	−7.28	_
*A* ^2^	24.67	0.0016	−16.18	_
*B* ^2^	0.37	0.5639	−1.97	_
*AB*	0.51	0.4988	3.06	_
Model	54.39	0.0001	_	_
Lack of fit	3.16	0.1475	_	_
Standard deviation	_	_	_	8.59
*R* ^2^	_	_	_	0.9749
Adj. *R* ^2^	_	_	_	0.9570

The model of fibers’ diameter obtained from experimental responses—based on regression coefficients—is shown in the following equation:

1 $$ Y = 208.18 + 47.15A - 7.28B + 3.06AB - 16.28A^{2} - 1.97B^{2} , $$

where *Y* is the fibers’ diameter, *A* is weight fraction of the polymer, *B* is the percentage of blended starch, and A*B is the interaction between weight fraction of the polymer and the percentage of starch.

**Fig. 2:**
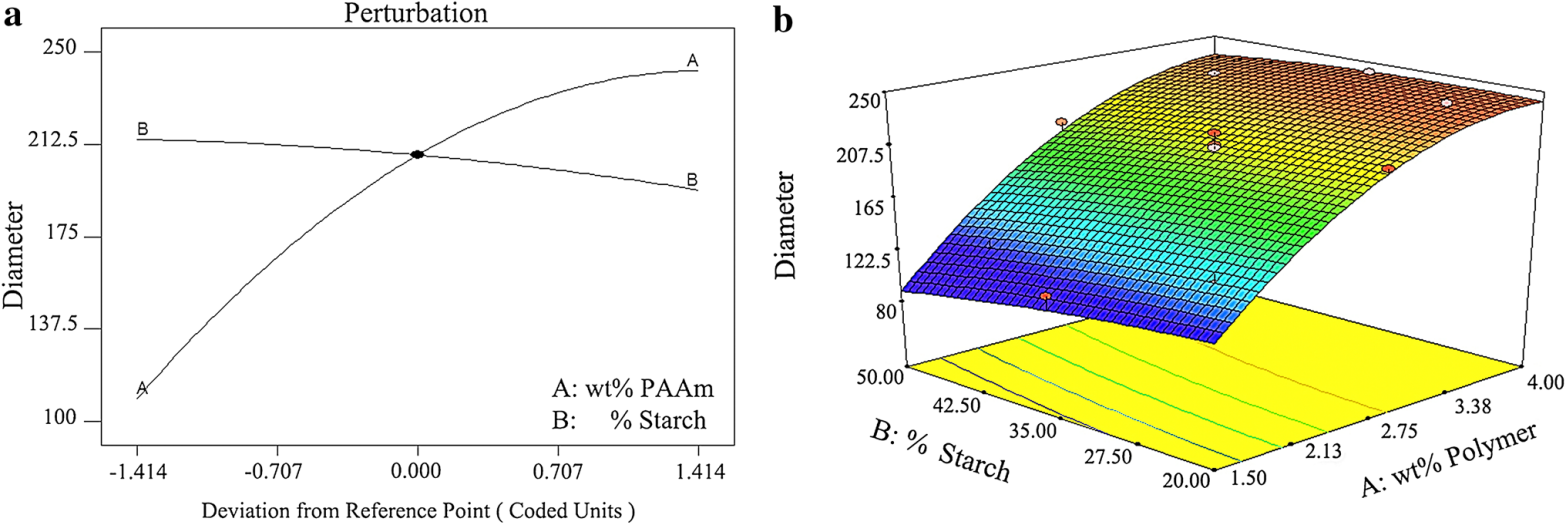
Nanofibers’ diameter as a response of wt% PAAm and % blended starch in **a** perturbation plot (**a**) and **b** 3D diagram

Perturbation plot represents the impact of percentage of the blended starch and weight fraction of the PAAm separately on the objective fibers’ diameter (Fig. 2a). It is clear that the diameter of nanofibers increases upon the weight fraction of PAAm increased. On the other hand, the diameter would slightly decrease upon the increment of the starch compounding percentage. Figure 2b shows the interaction between the weight fraction of the PAAm and the percentage of blended starch on the nanofibers diameter. This three-dimensional (3D) diagram indicates that increment in nanofibers diameter is affected by the augmentation of the PAAm weight fraction. Sharper slope of the curve in the side of weight fraction of the PAAm illustrates its dominant effect on diameter. Reduction effects of nanofibers’ diameter would be unfolded by the increment of the starch blending percentage with a constant weight fraction of PAAm.

#### FT-IR spectroscopy

**Fig. 3:**
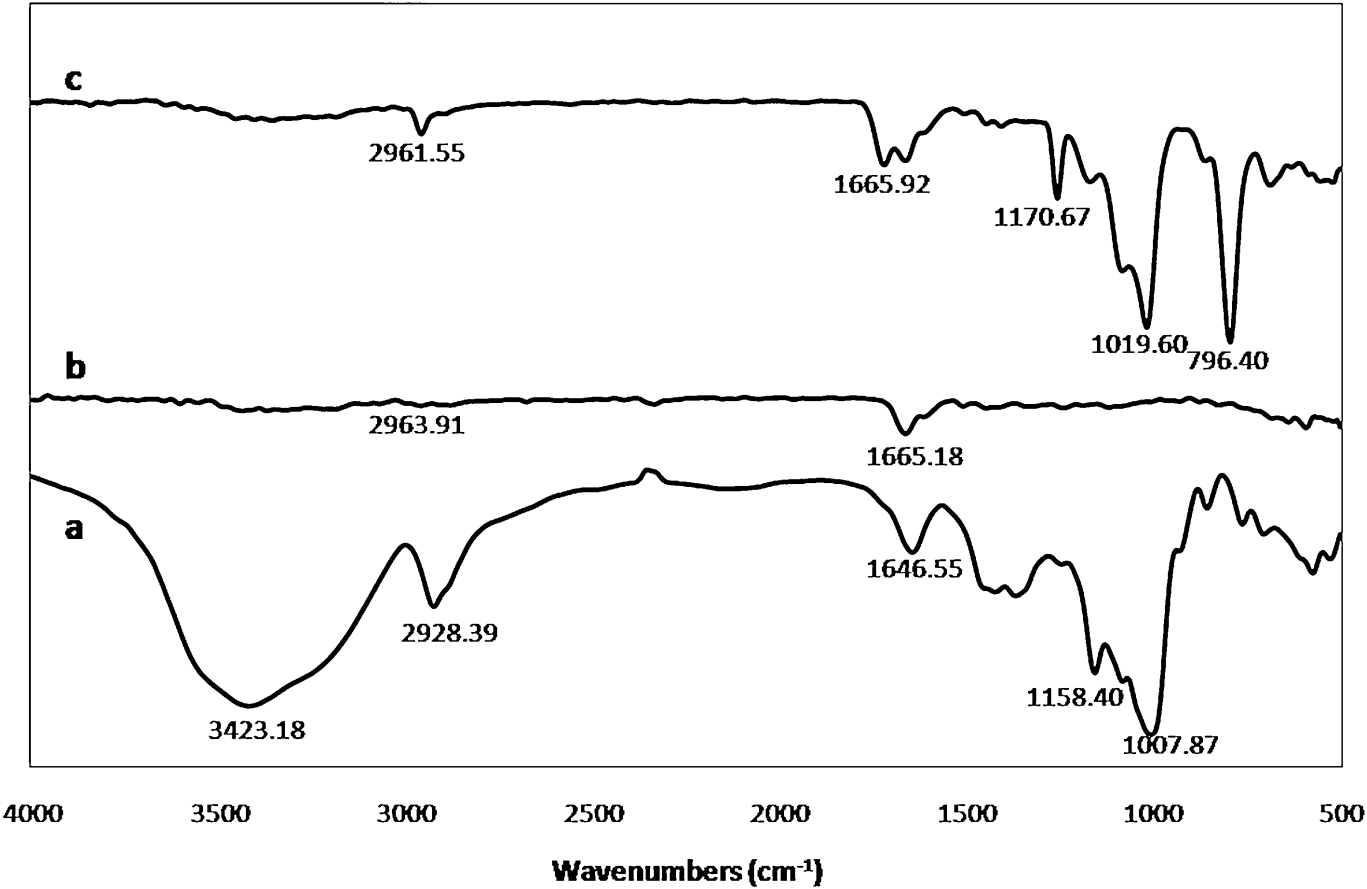
FT-IR spectra of *a* wheat starch, *b* PAAm nanofibers, and *c* PAAm/starch blend nanofibers

FT-IR spectroscopy is used to investigate the spectra of the wheat starch, PAAm, and blended PAAm/starch nanofibers, as shown in Fig. 3. IR Spectroscopy results were compared with the literature which showed a good agreement in closeness of peaks locations (Tang et al. 2008; Wang et al. 2011a; Zeng et al. 2011; Dwivedi et al. 2012; Liu et al. 2011; Soares et al. 2005; Sevenou et al. 2002; Majdzadeh-Ardakani et al. 2010). The peak reflecting C=O bonding for PAAm vibration at 1665.18 (cm^−1^) takes place at 1665.92 (cm^−1^) in the blended nanofibers (Tang et al. 2008; Wang et al. 2011a). The peak at 2963.91 (cm^−1^) is attributed to the vibration of N–H bending of PAAm in the nanofibrous format, which is presented at 2961.55 (cm^−1^) in the blended nanofibers.

In starch spectra, a strong and broad peak at 3423.18 (cm^−1^) is attributed to the characteristic absorption peak of the stretching vibration of O–H. The peaks at ~1084.07 (cm^−1^), 1158.40 (cm^−1^) and 1019.60 (cm^−1^) are related to the vibration of C–O in C–O–H groups. The peak at 2928.39 (cm^−1^) is ascribed to the asymmetric stretching of C–H whose intensity vibration is significantly lessened in the blend format. The peak at 1646.55 cm^−1^ is attributed to the adsorbed water. The peak at about 1007.87 (cm^−1^) is ascribed to C–O–H stretching. The peaks at 857 (cm^−1^) and 763.81 (cm^−1^) are related to the C–H absorbance of d-glucopyranosyl ring stretching.

Peaks at 1665.92 (cm^−1^) and 2961.55 (cm^−1^) imply the presence of PAAm, and peaks at 796.40 (cm^−1^), 1019.60 (cm^−1^), and 1170.67 (cm^−1^) are attributed to the starch in the blended nanofibers. FT-IR investigation demonstrates that blending was carried out in a satisfactory way due to the presence of all the starch and PAAm bonds in the blended nanofibers.

#### Thermogravimetric analysis

**Fig. 4:**
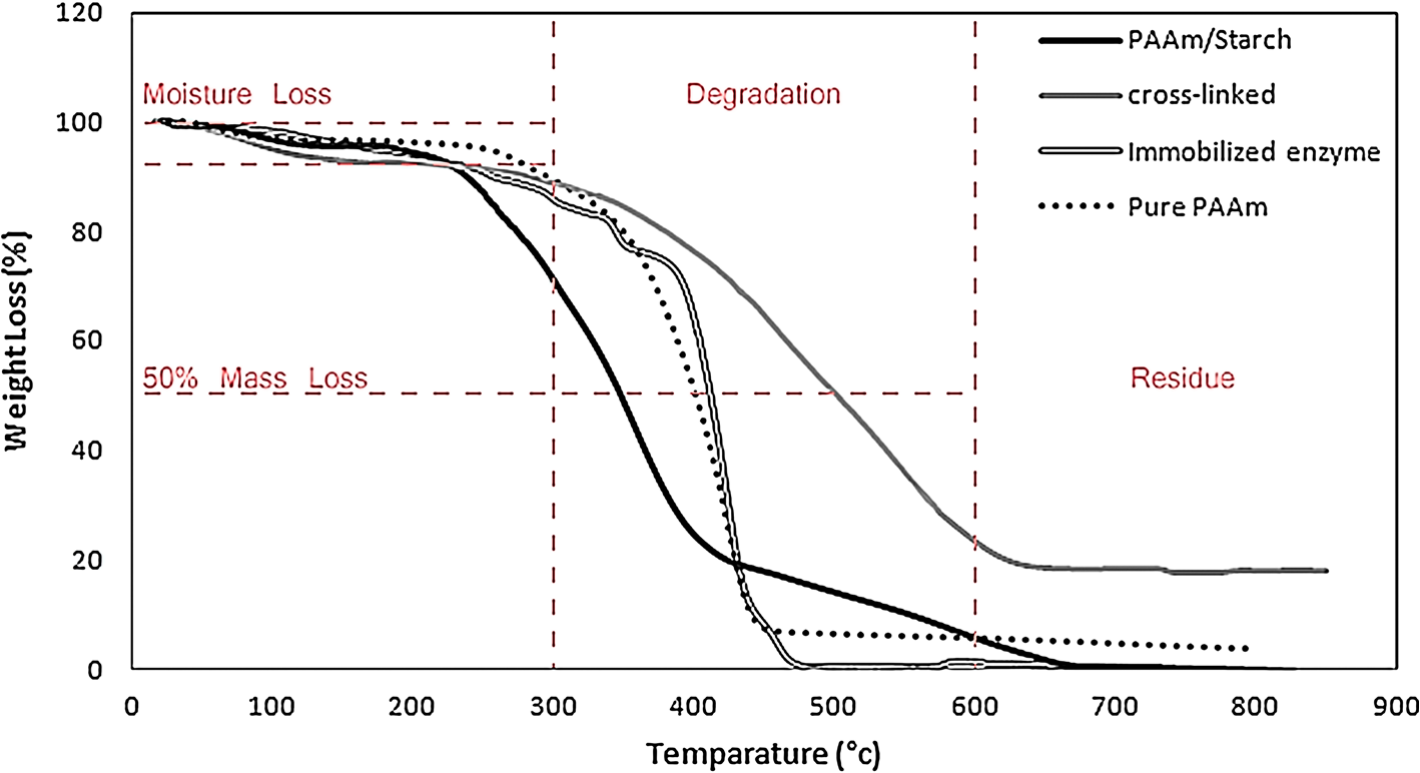
TGA curves of PAAm, 35% starch blended PAAm, cross-linked modified PAAm/starch, and phytase immobilized on PAAm/starch nanofibers at 2.75 wt% polymer solution

We characterize nanofibrous mats by thermogravimetric analysis (TGA) which is used to predict their thermal stability from the temperature of 25 °C up to 800 °C at heating rate of 10 °C/min under nitrogen atmosphere. Figure 4 shows the TGA curves of PAAm, blended PAAm/starch, cross-linked modified PAAm/starch, and also phytase immobilized PAAm/starch nanofibers. The curves show multistage degradation which demonstrates the decomposition of the starch and the PAAm in the blend format. Table 3 summarizes TGA analysis of the first derivative peak temperature (*T*_peak_), onset peak temperatures, 50% mass loss temperature, % mass change between 300 and 600 °C, and % residue of nanofibers weight loss. Observing the percentage of mass loss between 300 and 600 °C proves that the most part of degradation related to starch/PAAm decomposition is occurred in this range. The analyzed temperatures decreased with the addition of the starch loading in PAAm nanofibers, indicating a destruction in thermal stability because of the starch incorporation.

**Table 3:** TGA analysis results

Nanofibers	T50% mass loss (°C)	Inflect temp. (°C)	*T*_Peak_ (°C)	Mass change (%) between 300 and 600 °C	Residue (%)
Pure PAAm	401.5	310	430	83.86	3.57
PAAm/starch blend	348.41	207.4	360	65.48	0.5
Cross-linked blend	500	330	490	64.64	18.11
Blend with immobilized enzyme	411.5	361.3	425	84.47	0

Comparing mass loss temperatures of the PAAm/starch nanofibers and cross-linked ones can barely specify that the cross-linking modification significantly increases the nanofibers thermal stability. However, immobilization procedures and putting nanofibers in the aqueous enzyme solution for 18 h at 4 °C decrease the thermal stability (in comparison with cross-linked nanofibers). This is indorsed by comparing 50% mass loss temperatures (T50% mass loss) of the PAAm/starch nanofibers, cross-linked PAAm/starch nanofibers, and immobilized phytase on the PAAm/starch nanofibers, which are 348, 500, and 411 °C, respectively. Cross-linking improved thermal stability by about 43% measured at T50% mass loss and also it modified 18% mass residue (in comparison with blended nanofibers) which could be regarded as an evidence for proper cross-linking procedure. Nevertheless, holding nanofibers at 4 °C for 18 h while shaking in enzyme solution reduces thermal degradation of them (17.7% lower thermal stability compared to cross-linked nanofibers before immobilization).

According to TGA results, it is proved that the addition of starch reduces the thermal stability, since it increases the degradation of PAAm nanofibers as reported previously (Kaushik et al. 2010). A starch blended PAAm nanofibers of 2.75 wt% (containing 35% starch relative to PAAm) increases thermal degradation of pure PAAm nanofibers by 19.44%, in accordance with the first derivative peak temperature. Considering the intrinsic biodegradability of wheat starch, as thermal degradation of a polymer begins with chain cleavage, starch incorporation could lead to improving the thermal degradability of polymeric nanofibers according to the purpose which is using polymeric nanofibers in biological digestive application.

Decomposition kinetics of nanofibers can be inspected by observing the slopes of TGA curves of the PAAm and the blended starch ones. The sharper slope of the curve of the PAAm nanofibers indicates that its thermal decomposition is progressed at a higher speed than the blended nanofibers’ one which suddenly starts at 310 °C. Blended nanofibers originated from two components; hence, its thermal degradation behavior is a function of both starch and PAAm. Subsequently, it would result in a broad degradation peak and a lower speed.

#### Crystallography analysis

##### Differential scanning calorimetry analysis

**Fig. 5:**
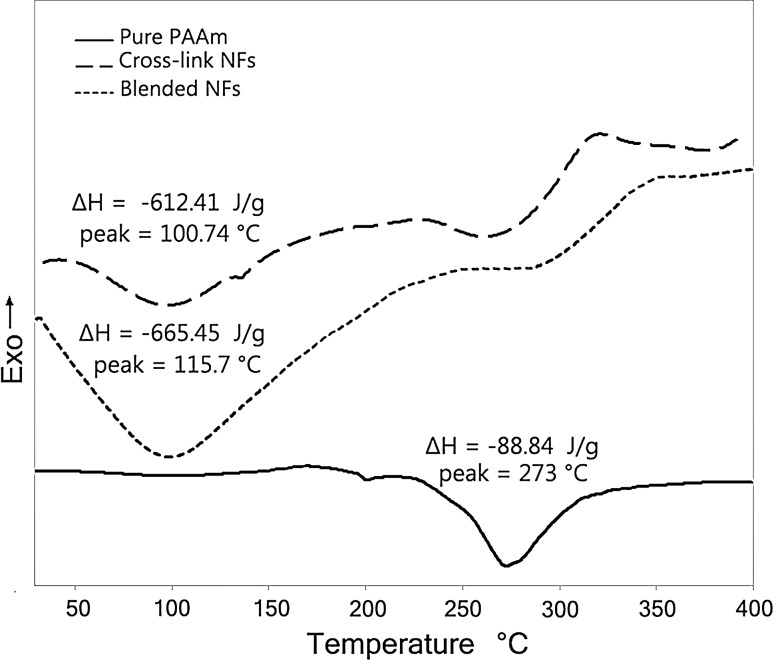
DSC curves of PAAm, 35% starch blended PAAm, and cross-linked modified starch/PAAm nanofibers at 2.75 wt% polymer solution

The exothermic DSC curves for pure PAAm, starch/PAAm, and cross-linked modified starch/PAAm nanofibers are shown in Fig. 5. It is obvious that different profile patterns appear after addition of the wheat starch.

Due to the slight change of the heat capacity, a weaker heat flow signal of the blended starch material is generated in comparison with other polymers (Liu et al. 2009). Therefore, measurement of glass transition temperature (*T*_g_) of the starch by DSC is difficult. Moreover, the multiple phase transitions during the heating process and the instability due to evaporation of water included in starch make the study of thermal behavior and crystallography of starch materials more difficult (Liu et al. 2009). Liu et al. evaluated *T*_g_ of starch containing different amounts of moisture by a high-speed DSC (above 50 °C/min) (Liu et al. 2009). Wheat starch DSC-related peak with different moisture occurs in the range of 60–105 °C which contains about 0.8% lipids and 28% amylase (Lionetto et al. 2005).

In pure PAAm, despite the fact that the peak centered at 273 °C, no significant melting point is observed due to the poor crystalline structure of PAAm which is proved by XRD analysis thereafter. In blended nanofibers, the presence of two peaks is observed and the rather huge exothermic peak centered at 100 °C is related to the starch and the peak which is shifted at 280 °C belongs to the PAAm (Amini et al. 2013; Lionetto et al. 2005; Zeng et al. 2011), although its conjunction shows the finest inter mixture of two phases and satisfactory compounding. The *T*_g_ of the pure PAAm nanofibers was measured at 189 °C. Disappearing of *T*_g_ in the X-linked blend nanofibers is the evidence of satisfactory cross-linking treatment of nanofibers. Both starch and PAAm peaks are sustained after X-linking with the difference of enthalpy (Δ*H*) of the starch caused by losing its moisture.

##### XRD pattern

**Fig. 6:**
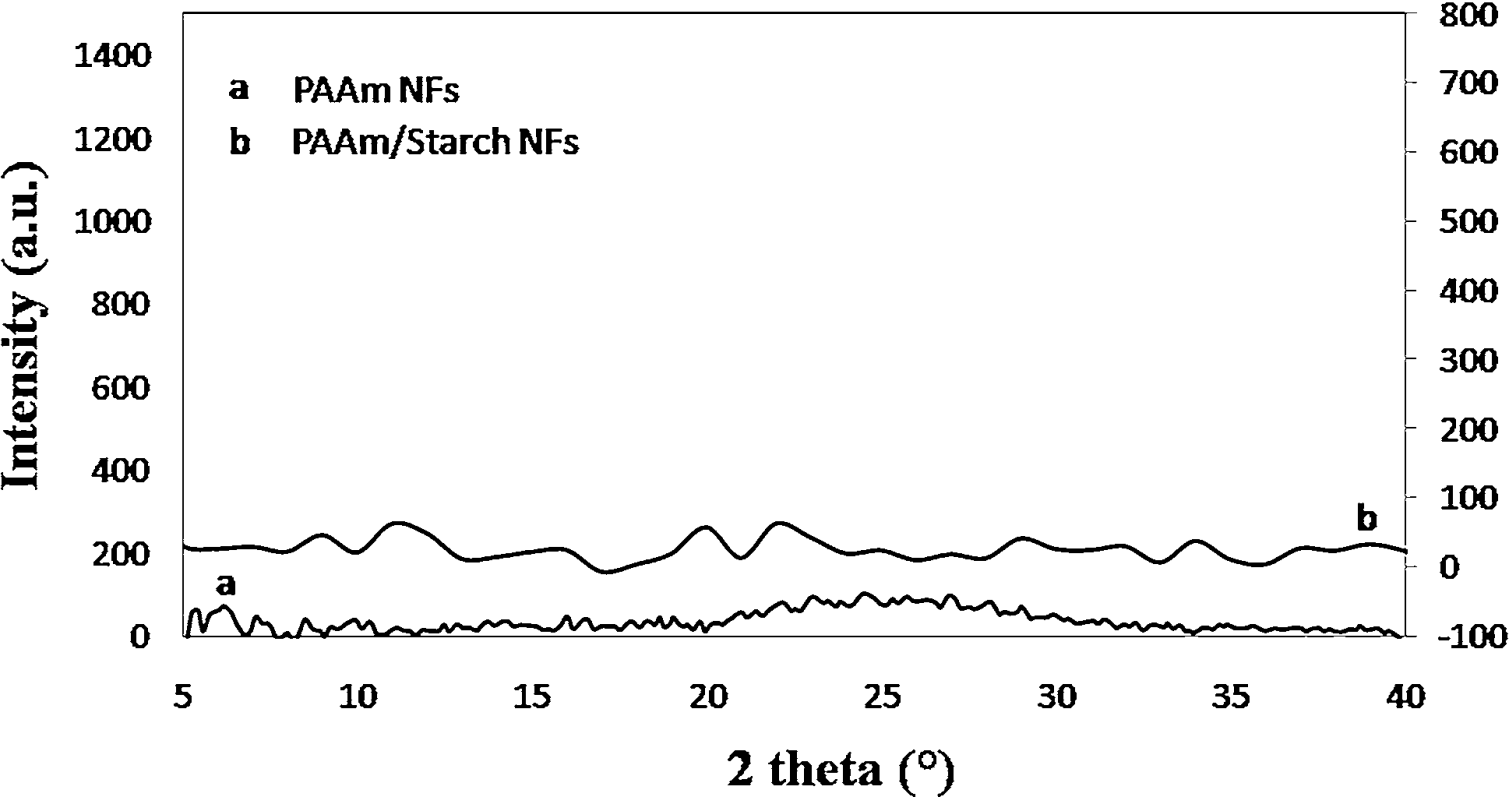
XRD patterns of PAAm nanofibers (*a*) and PAAm/starch blend nanofibers (*b*), respectively

To study the physical properties of the resultant fibers, it is very important to study the crystal structure of nanofibers made by PAAm and starch blended PAAm. XRD patterns of the pure PAAm and the blended starch/PAAm nanofibers are shown in Fig. 6. It was supposed that there should have been at least one crystalline peak due to the presence of the starch from the blended nanofibers (Wang et al. 2011b; Zeng et al. 2011; Lionetto et al. 2005). However, it can be observed that the blended nanofibers show a typical non-crystalline pattern. This can be justified by sample preparation and electrospinning conditions.

In accordance with the literature, pure PAAm nanofibers did not show any significant crystalline pattern over a 2*θ* range of 5°–40° (Tang et al. 2008). As reported in the literature, X-ray diffractograms of the wheat starch samples show typical of amorphous systems with sharp peaks centered at 2*θ* around 15°–18°–23° (Wang et al. 2011b; Zeng et al. 2011; Lionetto et al. 2005). Although, when it is blended with other polymers and electrospun to nanofibers, the crystalline structure of resultant fibers has been destroyed and the intensity of the peaks is decreased substantially (in comparison with its pure powder type). Moreover, followed by being solved in formic acid during solution preparation, the orientation of the starch’s chains may be disordered. Even more, it is observed that the amount of the starch in accordance with PAAm in the solution is a lot less. It is supposed that solvent rapid vaporization, electrical driving force, and stretching of fibers imposed by electrospinning procedure do not lead to orientated arrangement of the chains and crystalline structure (Khan et al. 2009).

It has been shown that neither type of fibers shows crystalline structure. Observing XRD patterns of both PAAm and starch blended PAAm nanofibers indicates that they have almost the same non-crystalline pattern. We hypothesize that there is no more crystalline phase existed; however, it may also be possible to claim that there might be less than 5 wt% from which it could be inferred that the dominant phase of samples is amorphous. Accordingly, it can be deduced that blending was successfully accomplished as the resultant nanofibers are in single phase.

### Biological assays

#### Immobilized enzyme activity measurements

**Fig. 7:**
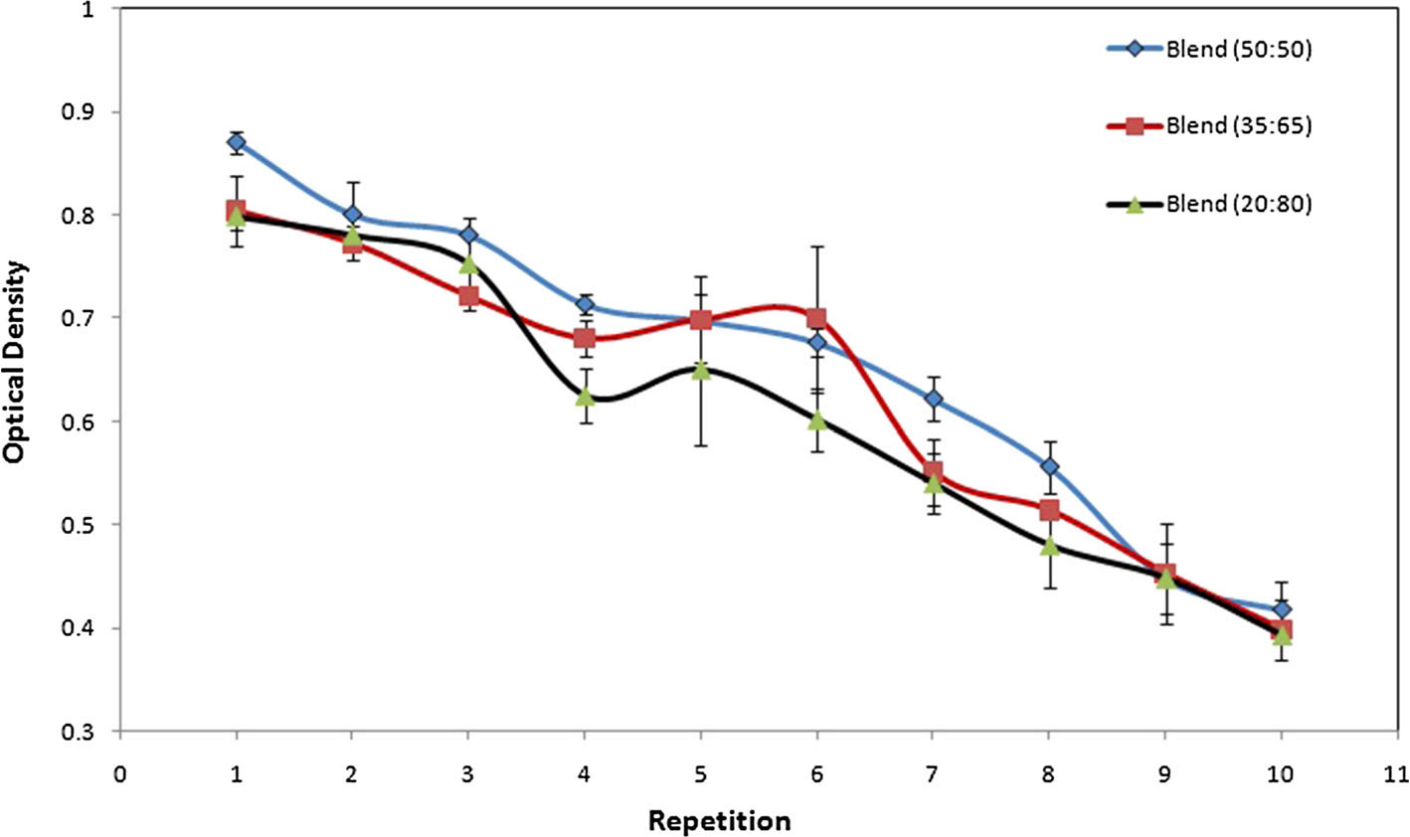
Remained activity of immobilized phytase

The effects of adding starch on the biological properties of PAAm nanofibers were investigated via the enzymatic measurements. Phytase is immobilized through covalent bonding by the creation of GA links on the surface of APTES-modified nanofibers and also concentration gradient of enzyme solution makes phytase migrate and get trapped inside the 3D structure of the PAAm/starch nanofibers. Starch incorporated nanofibers could accentuate biocompatibility and may facilitate absorption of phytase and increase activity. Figure 7 shows the optical density of assay mixtures for immobilized phytase on starch compounded PAAm nanofibers after 10 times of use, 2–3 times repetition at each assessment, to determine the remained activity. The immobilized enzyme on PAAm/starch nanofibers expresses more activity by increasing polymer concentration and percentage of the starch blending. At a constant weight fraction of the solution, increasing the amount of starch would affect the increment of the immobilized phytase activity. However, it is not completely effective to maintain its primary activity after ten times repetition. However, the optical density of the immobilized phytase on pure PAAm nanofibers is lower than the blended samples and it cannot maintain its activity and even its stability after 4th activity assessment. This demonstrates the effectiveness of the starch compounding for nanofibers activity and successful covalent attachment of phytase.

#### Kinetics investigation

**Fig. 8:**
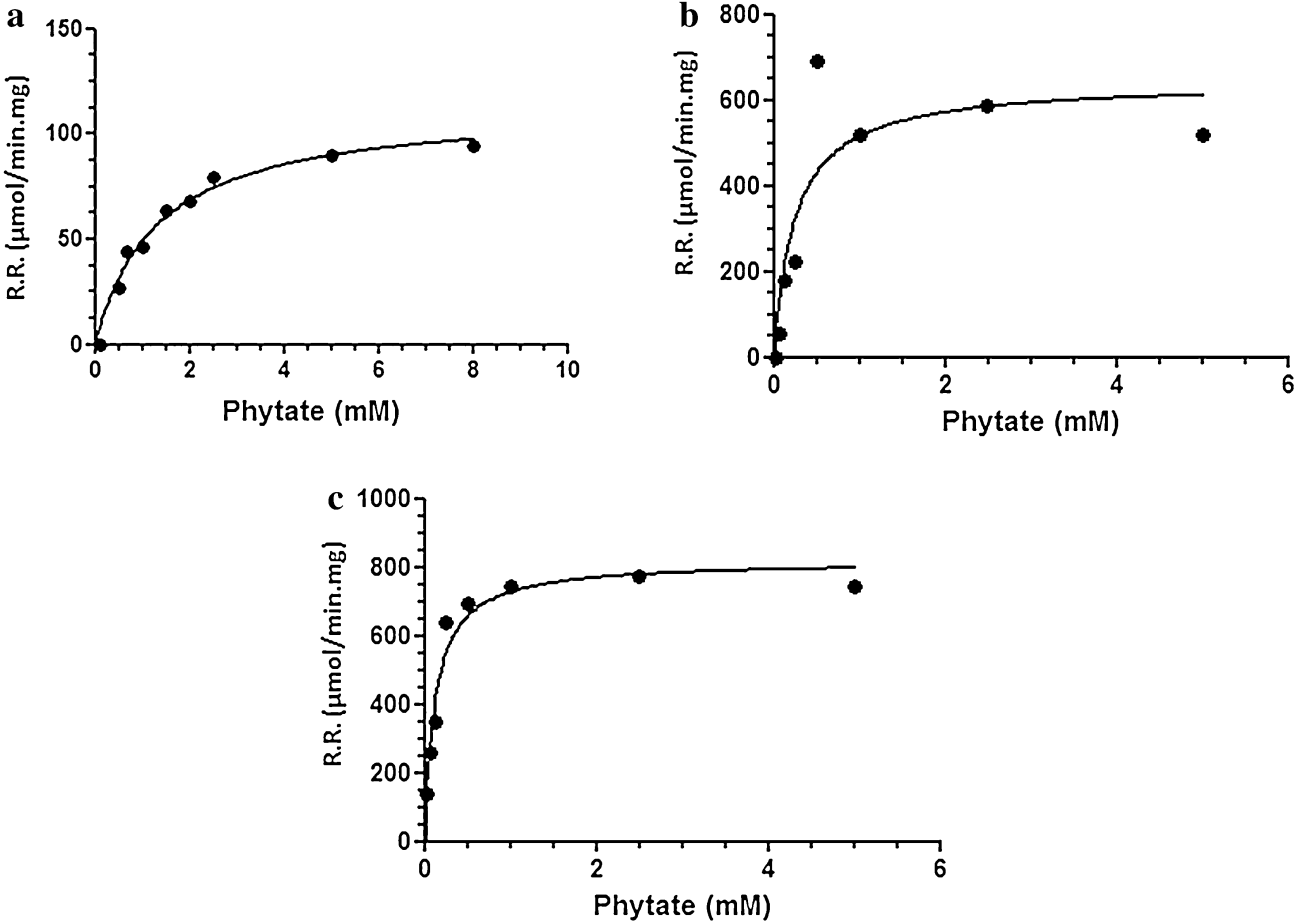
Michaelis–Menten curves for **a** free phytase, **b** immobilized phytase on PAAm nanofibers, and **c** immobilized phytase on PAAm/starch blend nanofibers

The investigation of the immobilized enzyme kinetics on the pure and the starch blended PAAm nanofibers and also in free condition was compared via Michaelis–Menten curves (Fig. 8). To evaluate *K*_m_ and *V*_max_, we obtained different concentrations of the substrate. Therefore, different appropriate solutions from phytic acid as a substrate of phytase were prepared. The assays were done under the same condition with three replicates for each concentration. The kinetic parameters are summarized in Table 4. The higher reaction rate and higher enzyme activity are represented by higher *V*_max_ and lower *K*_m_, respectively.

**Table 4:** Kinetic data for free and immobilized phytase

Enzyme type	*K*_m_ (mM)	*V*_max_ (µmol/min/mg)
Free	1.7	113
Immobilized on PAAm nanofibers	0.2445	642
Immobilized on PAAm/starch blend nanofibers	0.1214	816.6

Here, we report that the immobilization improves the kinetics of enzyme activity. In addition, it is proved that the presence of starch could slightly fix the reaction rate and it also provides improved activity in comparison with enzyme immobilization on pure PAAm nanofibers.

All biological assessments show satisfactory covalent attachment of the phytase. Besides, it is proved that the presence of appropriate amount of the starch could even repair biological properties.

## Conclusion

Here, we have synthesized polyacrylamide (PAAm), blended with different amounts of wheat starch and fabricated randomly oriented nanofibers. The SEM images and RSM diameter analysis have shown that more percentage of starch addition—proportionally against the percentage of PAAm—could lead to the reduction of the resultant nanofibers’ diameter. The blend was successfully made as the FT-IR results had proved the perfect structure. The thermal properties of PAAm nanofibers were shown to be improved by the presence of starch which enhanced the thermal degradability of PAAm nanofibers. Moreover, we have shown that cross-linking had synergetic effects in addition to surface modification which eventually enhanced thermal stability as well as preventing undesirable transformation; therefore, it increased the stability of nanofibers in unfriendly environment. This paper shows the promising potential of enzyme immobilization of starch/PAAm nanofibers. Overall, it was shown that the addition of starch could significantly increase the immobilized enzyme activity. We conclude that starch-aiding nanofibers improve kinetics activity of immobilized enzyme and also they surpassingly operate as a promising substrate for enzyme immobilization.
